# Thraustochytrids of Mangrove Habitats from Andaman Islands: Species Diversity, PUFA Profiles and Biotechnological Potential

**DOI:** 10.3390/md19100571

**Published:** 2021-10-14

**Authors:** Kaliyamoorthy Kalidasan, Nambali Valsalan Vinithkumar, Dhassiah Magesh Peter, Gopal Dharani, Laurent Dufossé

**Affiliations:** 1 Atal Centre for Ocean Science and Technology for Islands, National Institute of Ocean Technology, Dollygunj, Port Blair 744 103, Andaman and Nicobar Islands, India; vinith@niot.res.in; 2 Ocean Science and Technology for Islands, National Institute of Ocean Technology, Ministry of Earth Sciences, Government of India, Pallikaranai, Chennai 600 100, Tamil Nadu, India; mageshpeter@gmail.com (D.M.P.); dhara@niot.res.in (G.D.); 3 Chemistry and Biotechnology of Natural Products, CHEMBIOPRO, Université de La Réunion, ESIROI Agroalimentaire, 15 Avenue René Cassin, CS 92003, CEDEX 9, F-97744 Saint-Denis, Ile de La Réunion, France

**Keywords:** Andaman, mangroves, thraustochytrids, omega-3 polyunsaturated fatty acids, bioactivities

## Abstract

Thraustochytrids are the most promising microbial source for the commercial production of docosahexaenoic acid (DHA) for its application in the human health, aquaculture, and nutraceutical sectors. The present study isolated 127 thraustochytrid strains from mangrove habitats of the south Andaman Islands, India to study their diversity, polyunsaturated fatty acids (PUFAs), and biotechnological potential. The predominant strains were identified as belonging to two major genera (*Thraustochytrium, Aurantiochytrium*) based on morphological and molecular characteristics. The strain ANVKK-06 produced the maximum biomass of 5.42 g·L^−1^, while ANVKK-03 exhibited the maximum total lipid (71.03%). Omega-3 PUFAs such as eicosapentaenoic acid (EPA) accumulated up to 11.03% in ANVKK-04, docosapentaenoic acid (DPA) up to 8.65% in ANVKK-07, and DHA up to 47.19% in ANVKK-06. ANVKK-06 showed the maximum scavenging activity (84.79 ± 2.30%) while ANVKK-03 and ANVKK-10 displayed the highest antibacterial activity against human and fish pathogens, *S. aureus* (18.69 ± 1.2 mm) and *V. parahaemolyticus* (18.31 ± 1.0 mm), respectively. All strains were non-toxic as evident by negative blood agar hemolysis, thus, the thraustochytrids are suggested to be a potential source of DHA for application in the health care of human and fish.

## 1. Introduction

Thraustochytrids are a group of unicellular monocentric, oleaginous, non-photosynthetic, osmoheterotrophic, eukaryotic-stramenopile protists, belonging to the kingdom Straminipila, class Labyrinthulomycetes, family Thraustochytriaceae [1]. Taxonomic positions of thraustochytrids remain controversial. Thraustochytrids are often inaccurately referred to as microalgae [2]. The evolutionary relationship of thraustochytrids with heterokont algae and the usage of the term heterotrophic microalgae for marine thraustochytrids are strongly opposed [3].

Thraustochytrids are originally classified based on their morphological characteristics [4], under the class Oomycetes because of their biflagellated zoospores [5], and later based on other morphological features such as life cycles, ultra-structure, thallus formation, ectoplasmic net elements, phylogenetic analysis, polyunsaturated fatty acids (PUFAs), and carotenoid profiles. There are currently 11 genera with more than 35 species belonging to the family Thraustochytriaceae, of which 50% of species are under the genus of *Thraustochytrium* (15 species), *Oblongichytrium* (four species), *Althornia* (one species), *Japanochytrium* (one species), *Schizochytrium* (one species), *Ulkenia* (three species), *Aurantiochytrium* (three species), *Sicyoidochytrium* (one species)*, Parietichytrium* (one species), *Botryochytrium* (one species), *Monorhizochytrium* (one species), *Hondaea* (one species), and *Labyrinthulochytrium* (two species). Recently, the genus of *Oblongichytrium* [6] and *Althornia* [7] are removed from this family, but scientists still consider them under the thraustochytrid family. In future, there is a possibility of re-revising the thraustochytrids [8] under 11 genera viz., *Thraustochytrium* [9], *Japanochytrium* [10] *Schizochytrium* [11], *Ulkenia* [12], *Aurantiochytrium* [6], *Sicyoidochytrium, Parietichytrium*, and *Botryochytrium* [13], *Monorhizochytrium* [14], *Hondaea* [15], and *Labyrinthulochytrium* [16].

Thraustochytrids are abundantly present in marine and estuarine environments including water column, sediments, algae, mangroves, particulate detritus, and invertebrates [17,18]. These unicellular and heterotrophic protists are predominant in decomposing leaf litter in mangrove ecosystems [19]. The thraustochytrids adhere and penetrate the substrates with the support of ectoplasmic network, and they are able to produce a variety of hydrolytic enzymes such as amylase, protease, phosphatase, pectinase, lipase, esterase, cellulase, and xylanase [20]. These hydrolytic enzymes solubilize nutrients, which can be absorbed by the cells through ectoplasmic network. Thus, the thraustochytrids play a vital role in litter decomposition, nutrient enrichment, and food web enhancement in the mangrove ecosystem [17,21].

Thraustochytrids constitute a major component of the microbiota in mangrove ecosystems [22]. They are known as a promising microbial source for the commercial production of omega-3 polyunsaturated fatty acids (PUFA) such as docosahexaenoic acid (DHA), eicosapentaenoic acid (EPA), antimicrobial, antioxidant, and nanoparticle properties for their application in human health, aquaculture, and nutraceutical sectors [22,23,24,25,26]. DHA and EPA are also known to have positive results in the prevention of cardiovascular, neurological, and anticancer diseases [27,28]. The DHA is essential for new tissue formation, fetal neural development, visual sensory, brain development, and maintenance of brain function in human adults and for fish larval development. Hence, DHA is used in food products, drugs, and animal feeds [23,29]. Currently, fish and fish oils are major sources for the production of DHA. However, the fish oil contains a low level of DHA and large-scale production of the DHA from the fish oil is difficult in the context of the over-exploitation of fish stock. In this regard, thraustochytrids need to be considered.

Mangrove ecosystems are present in the intertidal areas of tropical and subtropical regions in the junction between marine and terrestrial ecosystems such as estuaries, lagoons, creeks, and bays [30]. The mangrove ecosystem is a ‘microbial paradise’, supporting a diverse group of microbes and play a major role in the transformation of organic matter into a nutrient supply to many organisms, and it acts as an intensive sink for CO_2_ [30,31]. Mangrove vegetation in the Andaman and Nicobar (A&N) Islands is spread both in seaward fringes and creek areas, with a total area of 604 km^2^ with only 3 km^2^ in the Nicobar Islands. The Islands have a high diversity of mangroves belonging to 13 families, 19 genera, and 38 species [32] and are located about 1200 km away from the mainland of India. They consist of a chain of 836 islands that include islets and rocky outcrops of which 38 are inhabited. These islands are rich marine biodiversity under erratic weather conditions, which make them unique from other geographic places. 

To the best of our knowledge, no study is available on thraustochytrids species in the Andaman and Nicobar Islands. Hence, this present work was undertaken to explore thraustochytrids from different mangrove habitats of the Andaman Islands and to assess their biological activities.

## 2. Results and Discussion

### 2.1. Morphological and Molecular Identification

**Figure 1:**
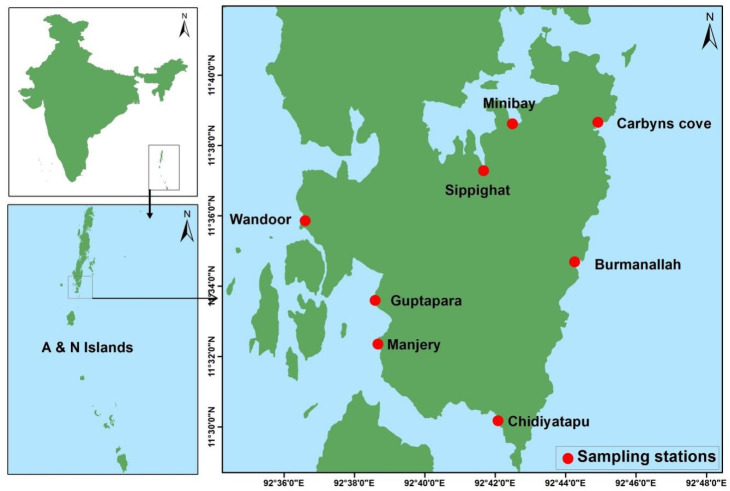
Map of south Andaman indicating sampling sites.

To understand the distribution and diversity of marine thraustochytrids, field surveys were undertaken from eight different locations of south Andaman mangrove habitats (Figure 1). Freshly collected mangrove leaf litter was used for isolation of thraustochytrids by direct agar medium incorporated with antibacterial (streptomycin 100 µg·L^−1^) and antifungal (Fluconazole 100 µg·L^−1^) agents to avoid contamination during the isolation process without causing any deleterious effect on the growth of thraustochytrids [19,25]. The direct plating method was used for isolation of thraustochytrids as it is extensively adopted [19,22,25], and pectin and glutamic acid also released from the decaying mangrove leaves induce a strong chemotactic response and attract the colonization of thraustochytrid zoospores [33]. In total, 127 pure cultures were obtained, but only 48 axenic cultures survived after three times of sub-culturing. 

PUFA-rich thraustochytrids were recorded at the maximum from Carbyns Cove station, which is situated in the northeastern part of the south Andaman with fresh water influence, sewage effluents, and the presence of high organic matter in the mangrove environment. The thraustochytrids were found to be low from Burmanallah, which is located in the northeastern part of the south Andaman, with clear waters, scattered dead coral reef, and a flat rocky shore in the mangrove habitat. Notably, *Schizochytrium* strains were isolated in Guptapara, which is a pristine mangrove habitat in the western part of the south Andaman. The presence of thraustochytrids have been reported only in limited areas of Indian mangrove regions such as Goa [34], Kerala [35], Mumbai [36], and Tamil Nadu [19,22,25,26]. Thraustochytrids for many mangrove habitats of global mangroves have still been poorly studied. The present work is a pioneer study on thraustochytrids from the A&N Islands.

After two to seven days of incubation, the thraustochytrid colonies displayed pale white, white, pale orange, orange in color, cream, oily with smooth and rough surface, looked small to larger colony size on the agar medium. Prolonged periods of incubation changed the colony color to orange from pale white, white, cream and oily colonies, whereas the colonies became colorless when incubated at 4 °C. The predominant thraustochytrid strains were examined under light (40X) and a scanning electron microscope for their size, shape of zoospores, and ectoplasmic network formation (Supplementary Figures S1–S12). The cell size ranged from 2 to 32 µm in diameter, and the cell sizes of *Thraustochytrium, Schizochytrium*, and *Aurantiochytrium* strains varied with their mode of reproduction, as reported by earlier works. *Thraustochytrium* reproduces sexually by zoospore formation [23], and its cell size remains to increase until sporangium is completely developed to release zoospores. *Aurantiochytrium* and *Schizochytrium* cells reproduce through the binary fission type of division and continue to divide at regular intervals [37], and these genera exhibit better growth and doubling time than *Thraustochytrium* because of their mode of cell division or reproduction [38].

**Figure 2:**
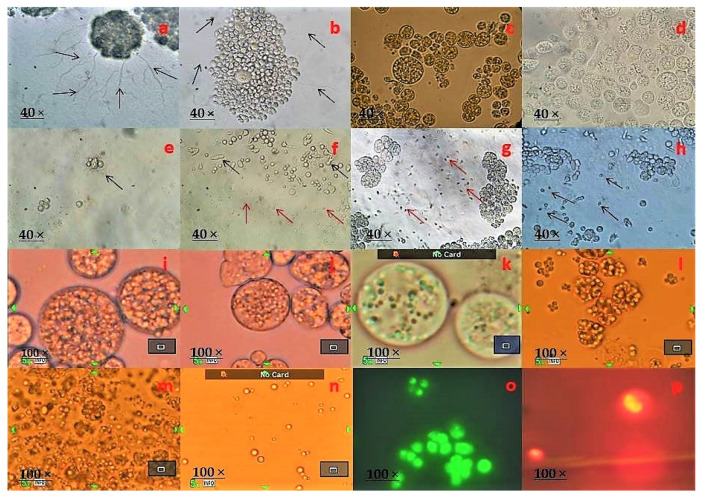
Developmental stages of thraustochytrids under light microscopic observation. (**a**) Clusters of vegetative cells dividing through binary cell division with ectoplasmic network, (**b**) Clusters of mature sporangium along with smaller vegetative dividing through binary cell division with ectoplasmic network and the presence of many small hair like filamentous structures, (**c**) Mature thallus with zoospores, (**d**) Mature zoosporangium with active zoospores as it is about to explode, (**e**) Vegetative cells and sporangiophores (just exploded in zoosporangium), (**f**–**h**) Spherical or pseudopodia shape of vegetative cells, different shape of amoeboid cells and many active zoospores with (**f**) globose, (**g**) reniform, (**h**) ovoid shape, (**i**) Mature thallus with granular content, (**j**) Many intercellular organelles such as Golgi bodies, vacuoles, lipids, and nucleus, (**k**) Mature zoosporangium with lipid globules and vacuoles, (**l**) lipid globules/droplets about to explode, (**m**) lipid globules fully exploded and dispersal over the culture medium, (**n**) individual lipid globules/droplets, (**o**) thraustochytrids acriflavin stained under an epi-fluorescence microscope, and (**p**) thraustochytrids stained with Nile Red under an epi-fluorescence microscope.

**Figure 3:**
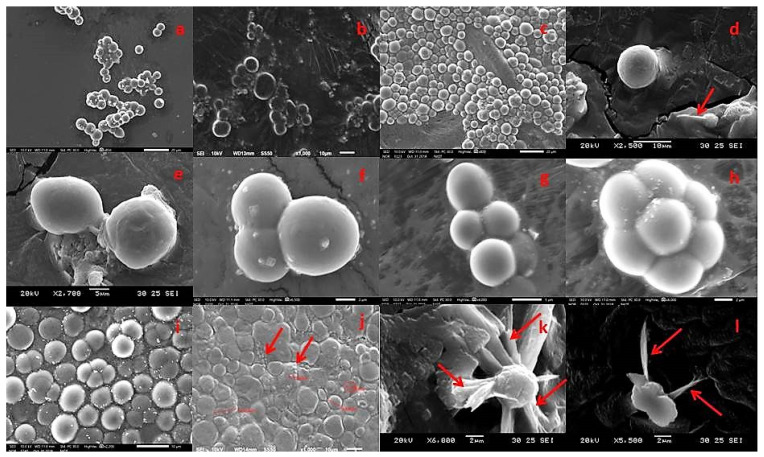
Developmental stages of thraustochytrids seen under scanning electron microscopic observation. (**a**) Vegetative cells in globose, (**b**) sub-globose, (**c**) spherical shape, (**d**) single mature vegetative cells and active zoospores, (**e**) vegetative cells dividing through binary cell division (diads), (**f**) group of three vegetative cells, (**g**) group of four vegetative cells (tetrads), (**h**) cluster of seven vegetative cells, (**i**) the vegetative cells can be seen any type of the growth of cells mature thallus, (**j**) larger amoeboid cells with reniform or ovoid shape zoospores, (**k**) The filaments of the ectoplasmic network, (**l**) zoospore with two heterokont flagella laterally inserted.

Thraustochytrid cells were observed to be globose and sub-globose in shape with different numbers of two to ten cells. During life cycle development, different shapes of cells including amoeboid, motile zoospores, heterokont flagella, ectoplasmic network, and exploding lipid droplets were observed (Figure 2 and Figure 3). The thraustochytrids were initially identified based on the acriflavine hydrochloride staining method and observed under epifluorescence microscope (Figure 2). This staining technique is used for rapid identification, but it is not specific to thraustochytrids, and it is also difficult to stain various shapes of cells such as amoeboid, zoospores, and young vegetative cells during life cycle by acriflavine hydrochloride [39]. As the staining technique is not very precise for morphological identification of thraustochytrids [40], the present study identified thraustochytrids based on intracellular lipid accumulation by using the Nile Red staining method [18] (Figure 2).

Thraustochytrids are identified based on morphology and life cycle characters [41], but it is very difficult to identify them at the species level. This is due to the many morphological characteristics that overlap with each other [6,42]. Therefore, 18S rRNA sequencing studies are important in understanding the taxonomy of thraustochytrids for rapid and accurate identification [13]. However, a major drawback is the non-availability of many reference 18S rRNA or complete gene sequence data in GenBank. 

The present work was attempted for molecular identification of 12 thraustochytrid strains using the 18S rRNA sequence. DNA was extracted from the strains and amplified using a primer specific for thraustochytrids. All the 12 thraustochytrids (ANVKK-01–ANVKK-12) isolates were amplified with the desired amplicon size (600 bp) and amplicon products were sequenced (SciGenome Labs Private Ltd., Cochin, Kerala, India) using the standard cycle sequencing protocol. The partial 18S rRNA sequence (NCBI BLAST) confirmed the systematic position of the isolates from 92.32% to 99.66%, similar to thraustochytrids group reported in NCBI, and nucleotide sequences were submitted to the NCBI Gene Bank database and accession nos. OK350759, OK350760, OK350761, OK350762, OK350763, OK350764, OK350765, OK350766, OK350767, OK350768, OK350769, and OK350770 (Table 1).

Based on the NCBI BLAST analysis, the marine thraustochytrids ANVKK-01 (accession no. OK350759) was found closer by 99.66% with *Aurantiochytrium* sp. TF49 (KM023693), 99.49% with *Aurantiochytrium* sp. UMACC-T022 (KP015192), and 98.82% with *Aurantiochytrium* sp. 18W-6a (AB811025). ANVKK-02 (accession no. OK350760) was 94.41% similar to *Thraustochytrium* sp. BP3.2.2. (DQ834732), 93.28% with *Aurantiochytrium* sp. B36 (MW629376), and 93.31% with *Aurantiochytrium* sp. B8 (MW629371). ANVKK-03 (accession no. OK350761) was found closer by 98.20% with *Aurantiochytrium* sp. LA22 (KY970084), 97.87% with *Aurantiochytrium* sp. LR52 (KY970085), and 97.23% with *Aurantiochytrium limacinum* (AB810939). ANVKK-04 (accession no. OK350762) was found closer by 97.58% with *Aurantiochytrium* sp. LA22 (KY970084), 97.25% with *Aurantiochytrium* sp. LR52 (KY970085)*,* and 96.79% with *Aurantiochytrium* sp. SY-52 (MT906356). ANVKK-05 (accession no. OK350763) was found closer by 97.23% with *Aurantiochytrium* sp. HS399 (MH319310), 97.23% with *Aurantiochytrium* sp. MBT-18 (MH488966), and 97.07% with *Aurantiochytrium* sp. CMFRIMBTDJMVL (MH059480). ANVKK-06 (accession no. OK350764) was found closer by 95.45% with *Aurantiochytrium* sp. B36 (MW629376), 95.29% with *Aurantiochytrium* sp. HS399 (MH319325), and 95.12% with *Aurantiochytrium limacinum* SL1101 (JN986842). ANVKK-07 (accession no. OK350765) was found to be closer by 92.32% with *Thraustochytrium* sp. BP3.2.2 (DQ834732), 90.98% with *Aurantiochytrium* sp. B8 (MW629371), and 90.65% with *Aurantiochytrium* sp. B31 (MW629374). ANVKK-08 (accession no. OK350766) was found closer by 92.82% with *Aurantiochytrium* sp. B31 (MW629374), 92.65% with *Aurantiochytrium* sp. B8 (MW629371), and 92.14% with *Aurantiochytrium limacinum* (MK056211). ANVKK-09 (accession no. OK350767) was found closer by 94.62% with *Thraustochytrium* sp. BP3.2.2. (DQ834732), 93.31% with *Thraustochytrium* sp. BP3.3.3. (DQ834737), and 92.98% with *Aurantiochytrium* sp. B36 (MW629376). ANVKK-10 (accession no. OK350768) was found closer by 94.11% with *Thraustochytrium* sp. BP3.2.2 (DQ834732), 92.80% with *Thraustochytrium* sp. B36 (DQ834737), and 92.13% with *Aurantiochytrium* sp. HS399 (MH319338). ANVKK-11 (accession no. OK350769) was found closer by 95.79% with *Aurantiochytrium* sp. B36 (MW629376), 95.45% with *Aurantiochytrium* sp. HS399 (MH319338), and 95.45% with *Aurantiochytrium* sp. UMACC-T025 (KR732620). ANVKK-12 (accession no. OK350770) was found closer by 94.81% with *Aurantiochytrium* sp. B8 (EU851172), 94.47% with *Aurantiochytrium limacinum* TWZ-H1 (EU851172), and 94.47% with *Schizochytrium limacinum* OUC192 (EU851172).

The BLAST results showed high 18S sequence similarity with respect to other thraustochytrid strain sequences available in the Genbank database. The high level of similarity suggests the ubiquitous nature of thraustochytrids. The phylogenetic tree was constructed including the available 18S rRNA thraustochytrid gene sequences of all our 12 isolates along with 108 18S rRNA various thraustochytrid sequences at the species level available in the Genbank database. The phylogenetic analyses confirmed the systematic positions of the thraustochytrids species from 92.32% to 99.66%. Topologically, the phylogenetic tree was comprised of three major clades, which were further subdivided into smaller clades. The maximum likelihood phylogenetic analysis showed a fairly complex consequence with robustly supported clades showing different species intermingling. Dellero et al. opined that two strains showing a full-length 18S sequence identity <92% could be assigned to different genera, while identities between 92% to 97% revealed congeneric species and identities above >97% confirmed the species level identification [15]. Based on these criteria, Andaman isolates ANVKK-01, ANVKK-03, ANVKK-04, and ANVKK-05 showing an identity (ID) above 97% and similarly query coverage above 98%, it was confirmed that the species level confirmation of thraustochytrid isolates such as *A. limacinum*. ANVKK-06 (95.45% ID), ANVKK-11 (95.79% ID), and ANVKK-12 (94.81% ID) showed an identity of 95%, however, it highly corresponded with *A. limacinum.* ANVKK-02 (94.41% ID), ANVKK-07 (92.32% ID), and ANVKK-10 (94.11% ID) was found to cluster together with *Thraustochytrium* sp. PB.3.2.2. (DQ834732), where this identity can be assigned to different/same genus and different species. ANVKK-08 showed 92.82% closer with *Aurantiochytrium* sp. B31 (MW629374), where this identity can be assigned to different/same genus and different species. 

**Figure 4:**
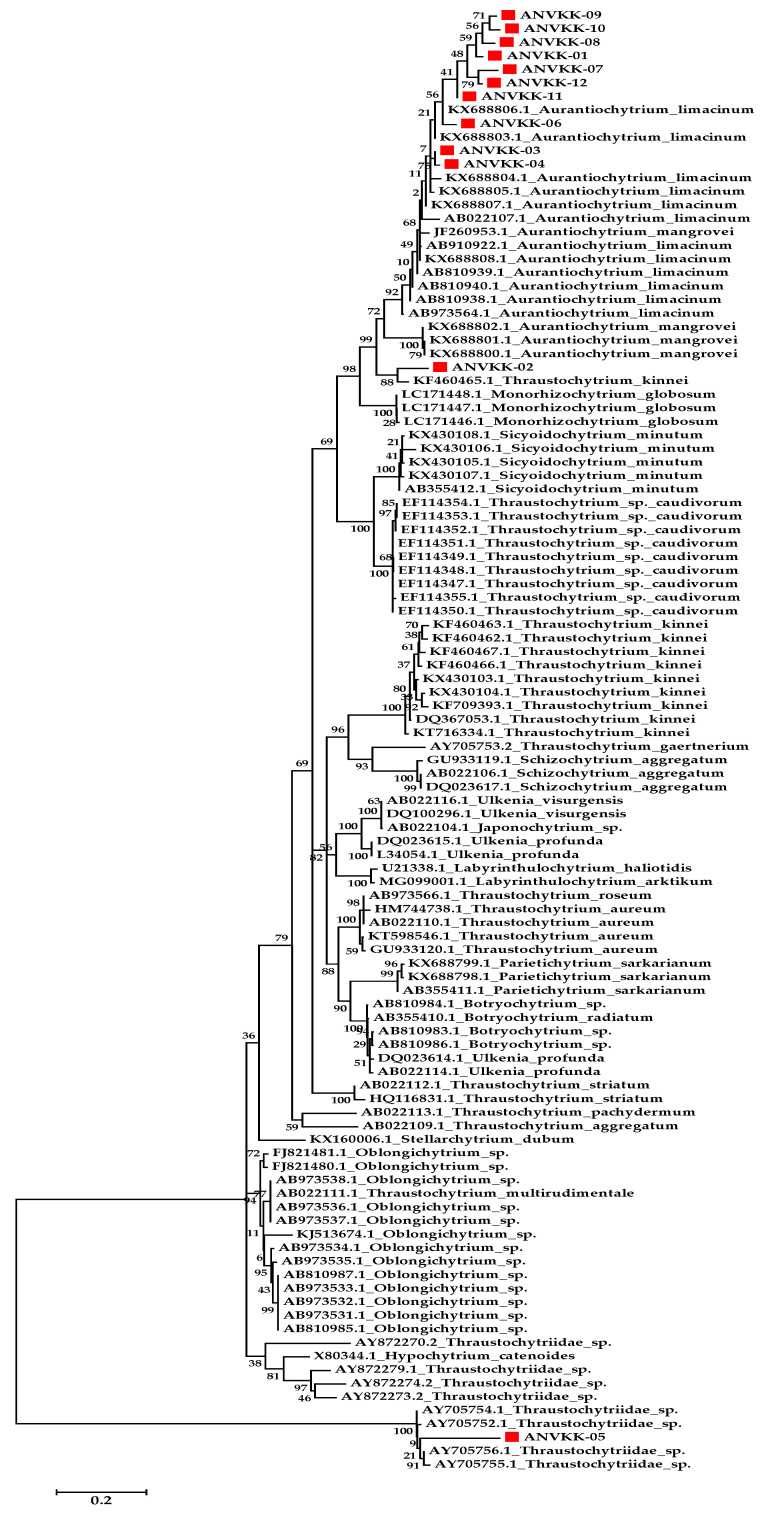
Molecular phylogenetic tree of thraustochytrid isolates compared with other related taxa. Branch distances represent nucleotide substitution rate and the scale bar represents the number of changes per nucleotide position. Thee red color circle and red color marking represent the thraustochytrids isolates (ANVKK-01-ANVKK-12) with the related GenBank submission numbers.

Based on the phylogenetic analysis of 18S rRNA sequences, 12 strains could be classified into two genera (i.e., *Thraustochytrium, Aurantiochytrium*). Isolate ANVKK-02 and ANVKK-05 were found to cluster together with *Thraustochytrium* sp. while the other ten samples were shown to cluster together with the *Aurantiochytrium* genus, particularly *Aurantiochytrium limacinum* (Figure 4). Both genera were reported for the first time in the mangrove habitats of the Andaman Islands, and *Aurantiochytrium* sp. was the most abundant species with ubiquitous distribution in most of the sampling stations, similar to other works [18,35,43]. The morphological and molecular features of all the isolates of Andaman thraustochytrids are given in Table 1. Our results are in accordance with the previous studies [6,35] where most of our isolates accumulated less than 1% of arachidonic acid. However, ANVKK-02 and ANVKK-05 did not accumulate arachidonic acid (Table 2), which is a basic property in the case of *Thraustochytrium* sp. The genus-level phylogenetic groups in the Labyrinthulomycetes can be distinguished by the combination of morphological and molecular features, although it is hard to distinguish each group based on a single feature. The major concern is the identification of thraustochytrids at the species level. The cultured diversity represents only a small fraction of their actual diversity and hence, there is a need for both novel culture methods and an uncultured diversity study on thraustochytrids.

### 2.2. Effect of Temperature, Salinity, and pH Tolerance

All of the isolated thraustochytrids were tested for growth at different ranges of pH (4–11), temperature (−4 °C to 50 °C), and salinity (0 PSU–100 PSU). All the isolates exhibited good growth in the pH range of 7–8, moderate growth at pH 6, and fair growth at pH levels of 4, 5, 9, and 10. Ten strains showed positive growth on almost all pH ranges (4–10), but, negative growth at pH 11. This reveals the ability of thraustochytrid strains to grow in acidic and alkaline conditions. Two strains (ANVKK-03 and ANVKK-10) showed negative growth at pH 4 and 10, and all other isolates had positive growth in a wide range of pH from 4 to 10. The pH range of 6.5 to 7.5 was found to be optimal for the growth of thraustochytrids (Table 2). Thraustochytrids are known to grow in a wide range pH from 4 to 9 [33,35], with better growth at neutral pH [44]. Being obligate marine forms, thraustochytrids prefer an alkaline pH [20] and the reduction in pH harshly affects the growth and biomass production in thraustochytrids [45].

Temperature is one of the most important factors to influence the growth of thraustochytrids. In the present study, growth of the thraustochytrids varied with different temperatures (10 °C to 35 °C) as good at 25 to 30 °C, moderate at 15 °C, 20 °C, and 35 °C, and fair at 10 °C. All the strains had negative growth at 5 °C, 40 °C, and 50 °C. ANVKK-03 showed no growth at 10 °C, and ANVKK-08 and ANVKK-11 exhibited moderate and fair growth at 15 °C (Table 2). Interestingly, thraustochytrids produced pigments at 10 °C. The growth and biomass production of thraustochytrids is generally affected at increasing temperatures [46]. Temperature plays a vital role in biomass and DHA production in thraustochytrids. Biomass production is reported to be high at high temperature, and in contrast, the amount of DHA increases at low temperature [47]. The maximum temperature tolerance was observed up to 35 °C in the present study, which is similar to previous studies [35,48]. However, thraustochytrids are able to withstand temperature changes (15–30 °C) on vegetative cell growth in culture conditions [33,49], similar to the habitat from where the strains are isolated.

All the thraustochytrids exhibited good growth at salinity ranging from 5 to 100 PSU. The strains of ANVKK-03, ANVKK-08, and ANVKK-10 showed moderate growth at 5, 90 PSU, and negative growth at 0 and 100 PSU. However, ANVKK-06 and ANVKK-07 exhibited fair growth at 100 PSU. In general, thraustochytrids showed good growth at 25 to 30 PSU, moderate growth at 10 to 20 and 35 to 70 PSU, and fair growth at 0 to 5 PSU and 80 100 PSU. Thraustochytrids are able to grow in a wide range of salinity (0–100 PSU), optimally at a range of 20–30 PSU. The salinity also plays a vital role in lipid accumulation [46], in accordance with the present results (Table 2). In general, all strains were found to be euryhaline, tolerating 0 to 100 PSU with good growth at high salinity and fair growth at lower salinity in accordance with earlier works [33,35]. However, ANVKK-03, ANVKK-08, and ANVKK-10 showed growth only above 5 PSU. Interestingly, many thraustochytrids strains were found to grow in zero salinity (0 PSU), but biomass and lipid production were significantly reduced, which is supported by earlier works on *Aurantiochytrium* [35], *Thraustochytrium* [50], and *Schizochytrium* [51]. Thraustochytrids are able to grow in a wide range of salinity (7.5–30 ppt) [33,49]. Generally, microbes isolated from mangrove and estuarine environment showed high salinity tolerance because of the highly fluctuating levels of salinity in the native habitats [22], similar to the present work. 

### 2.3. Growth Behavior of Thraustochytrids

**Figure 5:**
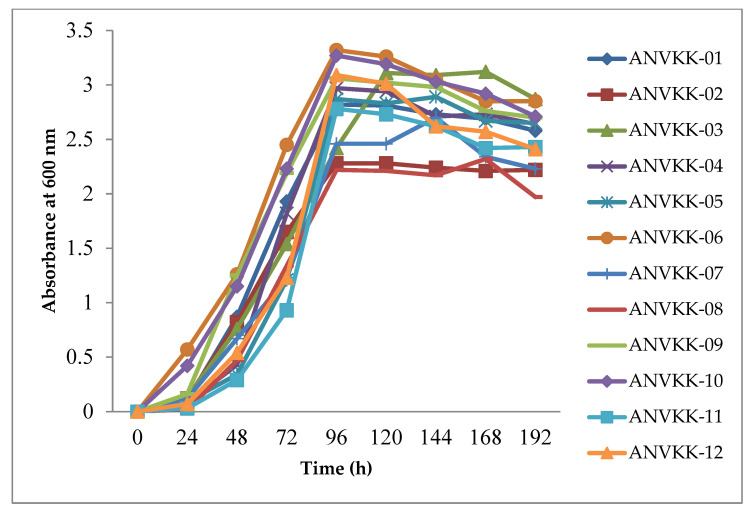
Growth curve characteristics of Andaman thraustochytrids isolates. The optimal culture conditions for screening the growth curve analysis were observed at 3 g·L^−1^ of glucose, 1.25 g·L^−1^ of yeast extract, and 1.25 g·L^−1^ of peptone, 7.2 pH, 28 psu salinity, 28 °C temperature, and eight days of incubation in 120× *g* maintained in an orbital shaker. The maximum growth was recorded in ANVKK-06 at 96 h.

Thraustochytrid growth was measured in terms of the optical density (OD) of the culture at 600 nm. The results showed that within 24 h of incubation, the growth was initiated in all strains except ANVKK-03. After 24 h of incubation, all strains showed significant growth during 48 h to 96 h and maintained their growth up to 120 h, but ANVKK-03 attained maximum growth at the end of 120 h. The cell death started from 144 h onwards for all strains except ANVKK-03 (168 h onwards) (Figure 5). Among the strains, ANVKK-06 showed the highest growth (OD value 3.32) and ANVKK-08 displayed the least growth (OD value 2.22). In general, the growth behavior of thraustochytrids varies with strains/species to strains/species and the results obtained in this study were similar to earlier work on thraustochytrids [35,50,52]. Generally, the growth of thraustochytrids depends on the availability of nutrients. Once the carbon source is depleted in the medium, the growth and cell division are reduced, whereas if the nitrogen source is depleted in the medium, the lipid is accumulated. Most of the thraustochytrids reach the stationary phase between seven to 10 days, with lipid bodies in vegetative cells [53].

### 2.4. Biomass Production of Thraustochytrids

**Figure 6:**
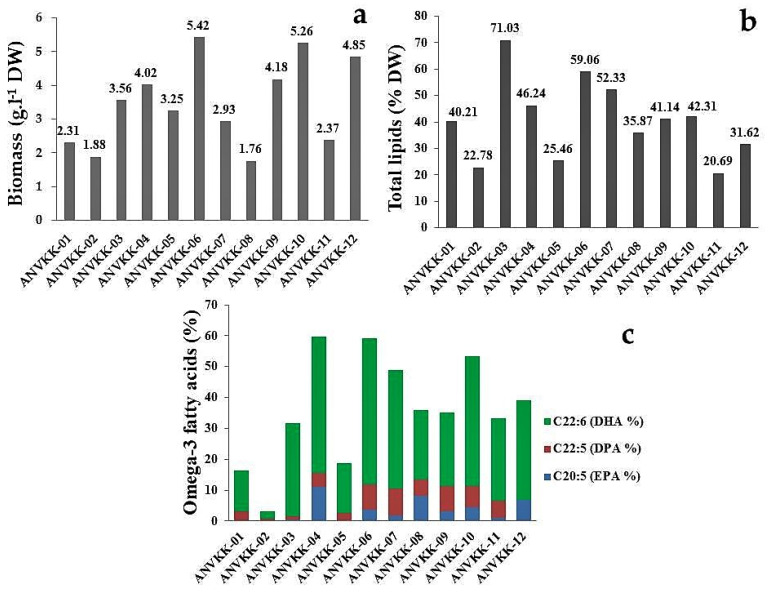
(**a**) Biomass, (**b**) total lipid, and (**c**) major omega-3 fatty acid production of the Andaman thraustochytrids isolates. The optimal culture conditions for biomass, total lipid, and omega-3 fatty acid production was observed at 7.2 pH, 28 psu salinity, 28 °C temperature, conducted up to nine days, 10 g·L^−1^ of glucose, 3 g·L^−1^ of yeast extract, and 5 g·L^−1^ of peptone. The maximum biomass productions were achieved on the fourth day of incubation.

All strains were observed as grains settling at the bottom of the culture flask and active zoospores floating over the surface or above the bottom. Biomass production varied from 1.76 g·L^−1^ to 5.42 g·L^−1^ dry cell weight, with maximum biomass of 5.42 g·L^−1^ produced by ANVKK-06 and the minimum biomass of 1.76 g·L^−1^ by ANVKK-08 with minimal carbon and nitrogen sources (Figure 6a). The optimal culture conditions for the highest biomass production were found to be 7.2 pH, 28 PSU salinity, 28 °C temperature, four days of incubation, 10 g·L^−1^ of glucose, 3 g·L^−1^ of yeast extract, and 5 g·L^−1^ of peptone.

The biomass production varies with species of thraustochytrids. *Schizochytrium* and *Aurantiochytrium* are fast growing compared to *Thraustochytrium* [54]. This is in accordance with the present work where *Schizochytrium* and *Aurantiochytrium* had higher biomass and lipids than *Thraustochytrium*. This is due to the mode of reproduction. Generally, growth in sexual reproduction is less than asexual reproduction. *Aurantiochytrium* and *Schizochytrium* cells reproduce through asexual binary fission whereas *Thraustochytrium* reproduces through sexual zoospore formation [5]. 

Carbon and nitrogen sources were used during biomass production in the exponential phase (48 to 72 h), which resulted in the maximum biomass production at 72 to 96 h. At the end of the stationary phase or extended phase, thraustochytrids utilize the lipid reserves for survival, which results in lower biomass and lipid production. During starved conditions, saturated fatty acids are consumed quickly, followed by PUFAs, therefore, carbon and nitrogen starvation should be avoided to achieve the maximum biomass and lipid production [55]. The presence of carbon and nitrogen sources in high proportion in thraustochytrid culture increases the synthesis of DHA [23]. In contrast, *Aurantiochytrium* and *Schizochytrium* are reported to consume carbon sources in the medium faster than *Thraustochytrium* to reach nitrogen starvation, which leads to faster lipid accumulation in thraustochytrid cells [56]. Hence, further studies are required to optimize the culture conditions to obtain the maximum biomass and lipid production.

### 2.5. Lipid and Fatty Acid Production

Lipid content varied from 20.69% to 71.03% of dry cell weight in 12 strains of thraustochytrids, with the maximum content (71.03%) in ANVKK-03 and the minimum (20.69%) in ANVKK-11. Six strains (ANVKK-03, ANVKK-04, ANVKK-06, ANVKK-07, ANVKK-09, and ANVKK-10) were found to be the high lipid producers with contents of 71.03%, 46.24%, 59.06%, 52.33%, 41.14%, and 42.31% of dry cell weight, respectively (Figure 6b). The major omega-3 PUFAs such as EPA (C20:5) was up to 11.03% in ANVKK-04; DPA (C22:5) was up to 8.65% in ANVKK-07; and DHA (C22:6) was up to 47.19% in ANVKK-06 (Figure 6c). In general, *Aurantiochytrium* and *Schizochytrium* are known to have high content of lipids and the level of fatty acids is reported to vary with strains/species and culture conditions. The major saturated fatty acid is palmitic acid (16:0), while the omega-3 polyunsaturated fatty acids such as EPA and DHA constitute 70 to 90% of total fatty acids [57].

Thraustochytrid species are a potential source for biodiesel and PUFA production compared to other microbes [54]. The present study found that the Andaman strains of thraustochytrids were rich in palmitic acid (SFAs), constituting 0.3% to 46.11%, especially in ANVKK-01 (46.11%), ANVKK-02 (39.71%), ANVKK-03 (41.05%), and ANVKK-12 (32.76%) (Table 2). The thraustochytrid strains that accumulate high levels of palmitic acid are considered as the best renewable fatty acid source for biodiesel production whereas omega-3 fatty acids are useful for nutraceutical potential [58]. The presence of arachidonic acid (C20:4) ranged from 0 to 3.94%. ANVKK-02 and ANVKK-05 did not accumulate arachidonic acid. Most of our isolates accumulated less than 1% of arachidonic acid, similar to earlier works that have reported less than 5% of arachidonic acid in *Aurantiochytrium* [6,35] 

EPA content varied from 0 to 11.03% and the thraustochytrid strains rich in EPA were ANVKK-04 (11.03%), ANVKK-08 (8.16%), and ANVKK-12 (6.92%). DPA varied from 0 to 8.65% with high DPA content in ANVKK-06 (8.21%), ANVKK-07 (8.65%), ANVKK-09 (8.01%), and ANVKK-10 (6.89%). DHA, the major omega-3 fatty acid, was present in all strains in a range from 2.37 to 47.19% and DHA was found to be high in ANVKK-06 (47.19%), ANVKK-04 (44.07%), ANVKK-07 (38.29%), and ANVKK-10 (42.14%) (Table 3). Our strain ANVKK-06 produced a higher level of DHA (47.19%) than the commercial strain of *Schizochytrium* sp. SR21 (30% DHA) [53]. The thraustochytrids were earlier reported to produce DHA in the range of 22 to 60% of total fatty acids [43]. The fast growing *Schizochytrium* species accumulates lipids up to 80% of dry cell weight compared to slow growing *Thraustochytrium* species [49]. The lipid and fatty acid production mainly depend on the availability of carbon and nitrogen sources [54,56]. Generally, lipid accumulation rises once the nitrogen source is exhausted in the culture medium. Lipids accumulate in thraustochytrids during the growth phase [53]. The biomass and lipid production was the maximum at the early stationary phase (four to five days), and prolonged incubation of thraustochytrids utilized the reserve lipid, resulting in negative lipid production.

DHA production in our thraustochytrid strains was high and it was also comparable with other commercial DHA producers. The fatty acid profile of thraustochytrids can be attributed to the habitat and the environmental factors from where they have been isolated. Therefore, culture optimization is further needed for maximum lipid and fatty acid production to improve the commercial scale [56].

### 2.6. Blood Agar Hemolysis Activity

**Figure 7:**
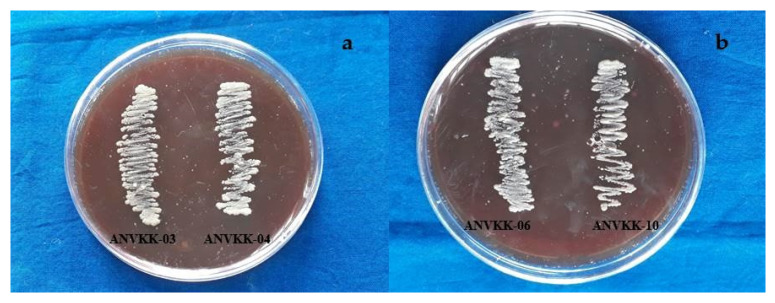
Blood agar hemolytic activity of Andaman thraustochytrid isolates. (**a**) ANVKK-03 and ANVKK-04, (**b**) ANVKK-06, and ANVKK-10 showed the absence of clear zone and color transparency around the colonies revealed that there was no blood lysis activity.

Thraustochytrid strains were tested for blood agar hemolysis against the goat blood sera. The inoculated Petri plates were kept for incubation at 30 °C for 24 to 48 h. After incubation, the plates were observed for the zone of clearance around thraustochytrid colonies. However, the absence of clear zone and color transparency around the colonies revealed that there was no blood lysis activity of thraustochytrids (Figure 7). This is called gamma hemolysis, meaning that there is no reaction in the medical microbial term. Several researchers have studied the hemolytic property of bacteria, but to our knowledge, this is the first study used to test for the toxicity of thraustochytrids.

*Staphylococcus aureus* is able to lyse goat blood and is called alpha hemolysis, hence the present study used it as a positive control as it showed a good zone of clearance and color transparency around the colony. All 12 thraustochytrids showed negative hemolytic results against goat blood cells and did not have pathogenic properties (Table 2). The common clinical pathogens and methicillin resistant *S. aureus* exhibited beta hemolytic lysis [59]. Most previous researchers have reported that Gram-negative bacilli bacteria and biosurfactant producing bacteria exhibit alpha hemolysis activities [60,61]. A pathogenic *Acinetobacter* exhibits beta hemolytic activity [62]. Clinical bacterial isolates are reported to have hemoagglutination and hemolytic activity against different human blood groups [63]. This study proved that thraustochytrids are non-toxic to blood cells and hence future clinical trials can be attempted for animal and human consumption or bio-product development.

### 2.7. Antibacterial Activity

The petroleum ether extracts of the thraustochytrids were tested against five human and four fish bacterial pathogens. The results are summarized in Table 3. ANVKK-03 and ANVKK-10 showed high inhibition against human and fish pathogens of *S. aureus* (18.69 ± 1.2 mm) and *V. parahaemolyticus* (18.31 ± 1.0 mm), whereas ANVKK-07 and ANVKK-01 exhibited low inhibition against human pathogens of *E. coli* (4.32 ± 0.5 mm), followed by *V. cholera* (4.34 ± 0.2 mm), and ANVKK-02 and ANVKK-12 also displayed minimum inhibition against fish pathogens of *V. alginolyticus* (4.15 ± 0.4 mm), followed by *V. harveyi* (4.66 ± 1.12 mm) (Table 4). This is supported by earlier works where thraustochytrid-derived fatty acids have antibacterial activity besides [25] antioxidant [26], antiviral [64], and anticancer [28] properties.

Mangrove associated marine fungi are a promising source of antimicrobial substances [65]. In the present study, petroleum ether extracts of thraustochytrids exhibited antibacterial activity against the human and fish bacterial pathogens, and this activity can be attributed to the presence of PUFAs in the extracts, in accordance with earlier reports with other species of thraustochytrids [19,22,35]. Fatty acids, especially DHA and EPA, play a key role for antimicrobial food additives, which inhibit the growth of unwanted microbes and have bactericidal activity against pathogenic microbes of *S. aureus*, *B. subtilis*, and *Pseudomonas aeruginosa* [66,67]. The ethyl acetate extract of mangrove-derived *Trichoderma* exhibited a wide spectrum activity against human and fish pathogens, and the highest activity was against *E. coli* (19 mm) and *V. harveyi* (20 mm) [68].

The antibacterial activities of thraustochytrids are advantageous in using them as probiotics in aquaculture to protect the host against pathogens. Further research on the petroleum ether extract for its purification and characterization will lead to the development of potential antibacterial drug. 

### 2.8. Antioxidant Activity

**Figure 8:**
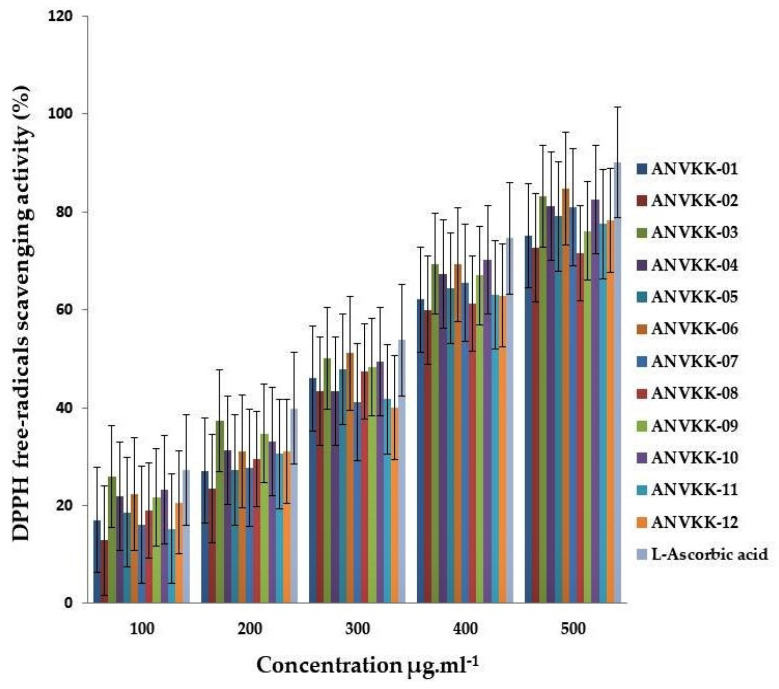
DPPH free-radicals scavenging (%) activity in extracts of Andaman thraustochytrids at different concentrations.

The antioxidant activity of the methanol extract of 12 thraustochytrids was evaluated by the DPPH radical scavenging assay and compared with L-ascorbic acid, which was used as the positive control. The results are shown in Figure 8. The DPPH free-radical scavenging assay was significant between thraustochytrids and the scavenging activity also increased with an increase in the concentrations of their extracts (*p* < 0.05) and this is in agreement with an earlier report on other species of thraustochytrids [26,69]. The activity was visually noticeable as the color changed from purple to yellow. ANVKK-06 exhibited the highest radical scavenging activity (84.79 ± 2.30%); whereas ANVKK-08 and ANVKK-02 showed a low scavenging activity of 71.65 ± 1.00% and 72.72 ± 1.15%, respectively (Figure 8). 

The thraustochytrids are a potential source of natural antioxidants and free radical scavengers due to the presence of omega-3 fatty acids [26]. The DPPH assay is an important tool for assessing antioxidant activity. The present study observed the maximum DPPH radical scavenging activity with *Thraustochytrium* and minimum with *Schizochytrium*, which is in accordance with earlier reports on marine thraustochytrids [26,69].

Omega-3 PUFAs have an important dietary role as antioxidant and chemotherapeutic agents [70]. The microalgal PUFA extracts were found to have antioxidant and dietary activities [71]. The present work found that PUFA rich thraustochytrids were high in antioxidant and free radical scavenging activities with potential applications such as health products, aquaculture nutrition, and human health products [19]. This antioxidant activity is attributed to enzyme activity and a reduction in lipid peroxides connected with oxidative stress resistance in various human diseases. 

The PUFA rich thraustochytrids can be explored for the production of a wide range of bioactive compounds as a supplement for preparations of animal feed [72], antimicrobial [19], antioxidant [26], biodiesel [73], enzymes [5], carotenoids [74], squalene [75], exopolysaccharides [76], vaccines [64], cancer drugs [28], and nanoparticles [22]. 

## 3. Material and Methods

### 3.1. Sample Collection and Processing

The decomposing mangrove leaves were aseptically collected from the intertidal mangrove habitats of the south Andaman. The leaf samples were transported to the laboratory and stored at 4 °C until further processed. The samples were washed with sterilized 100% natural seawater (NSW, 28 psu) with the addition of antibacterial (streptomycin 100 µg·L^−1^) and antifungal (Fluconazole 100 µg·L^−1^) agents to prevent contamination. The samples were then cut aseptically to 0.5 cm^2^ pieces and transferred to a Petri dish containing NSW with antibacterial and antifungal agents.

### 3.2. Isolation and Maintenance of Thraustochytrids

Four sample fragments were placed on a culture medium containing glucose (10 g·L^−1^) yeast extract (1.25 g·L^−1^), peptone (1.25 g·L^−1^), and agar (10 g·L^−1^) (GYPA) in natural seawater with the addition of antibacterial (streptomycin 100 µg·L^−1^) and antifungal (fluconazole 100 µg·L^−1^) agents to prevent contaminations, adjusted to the pH of 7.2 and incubated at 28 °C for 48 h. After incubation, the plates were examined daily up to seven days under a Nikon Eclipse Ni-U microscope (Nikon Corporation, Tokyo, Japan) set to bright field illumination. Once thraustochytrid colonies were found, they were then sub-cultured into fresh culture medium aseptically. These plates were then checked for growth and contamination daily. The pure isolates were maintained on agar slants, and sub-cultured once in 30 days.

### 3.3. Morphological Identification of Thraustochytrids by Light Microscope (LM) and Scanning Electron Microscope (SEM)

Thraustochytrids were morphologically identified based on color and shape during life cycle development such as the presence of ectoplasmic network, zoospore production, amoeboid cells, and vegetative cells. Pure colonies of thraustochytrid strains were taken from freshly sub-cultured glucose yeast peptone agar medium (GYPA) by using a sterilized inoculating loop, poured in a sterile slide chamber. These were examined after acriflavine staining at a concentration of 0.02 µg.mL^−1^ under the light microscope for morphological characters of thraustochytrids [13].

Further analysis was made using a scanning electron microscope (SEM). Briefly, the freeze-dried cells of thraustochytrids were placed on a glass slide, fixed with 2.5% *v/v* glutaraldehyde at 4 °C for 120 min, and washed three times with 0.1 M sucrose solution in 0.1 M cacodylate buffer, pH 7.2 for 5 min each. The specimen was post-fixed with 2% osmium tetroxide (OsO_4_) at a retention time of 90 min under the flow-hood, and then washed three times with distilled water for 5 min each. After fixation, the samples were dehydrated with ethanol at different concentrations (10, 20, 40, 60, 80, 90, and 100%) followed by substitution with isoamyl acetate. The mounted samples were transferred to carbon tape on an aluminum stub and dried by CO_2_. After applying a surface gold coating, the samples were observed under SEM (JSM-IT500, JEOL InTouchScope™, Tokyo, Japan).

### 3.4. Nile Red Staining

Lipid and fat globules were identified through Nile Red staining methods. The stain was prepared at a 1 µg/mL concentration in acetone. Fresh cells were taken from culture plates through a sterile inoculation loop and a cell was spread on a glass slide. A drop of Nile Red-acetone solution was then mixed with a drop of thraustochytrid cell suspension on the glass slide for staining and incubated for 2–5 min. This was washed with double distilled water to remove the excess of extracellular dye, and the cells were observed under epi-fluorescence microscope (Zeiss AxioSkop-2 PlusMercury-HBO50 ACL1, Carl Zeiss Microscopy, Oberkochen, Germany) with an epi-illumination using a high-pressure mercury light source at the wavelength of 450–500 nm with a pass exciter filter. The thraustochytrids with a golden yellow color fluoresced from the cells that corresponded to the presence of high intracellular lipid, and lipid globules in the cells were selected for further fatty acid analysis [18].

### 3.5. Genomic DNA Extraction, Sequencing, and Phylogenetic Analysis

The selected thraustochytrid strains were cultured in fresh GYP broth (glucose 3 g·L^−1^, yeast extract 1.25 g·L^−1^, and peptone 1.25 g·L^−1^) medium, supplemented with streptomycin and fluconazole 100 µg·L^−1^ at 28 °C, 7.2 pH, 28 PSU salinity, and 120× *g* for 48–72 h. After incubation, the cell culture was harvested by centrifugation (8000× *g* for 5 min at 4 °C) and washed twice with sterile distilled water to remove the medium constituents. Extraction buffer was added to the cell pellet (0.2 M Tris-HCl, pH 8.0, 1.4 M NaCl, 0.1 M EDTA, and 1.5% SDS) and kept in a water bath at 55 °C for 2 h. This solution was then centrifuged at 10,000× *g* for 5 min and the supernatant was used to extract the genomic DNA. Total genomic DNA was extracted from the washed cell pellet using the phenol-chloroform method, and the mixture was centrifuged at 10,000× *g* for 5 min., the aqueous phase was precipitated using ice-cold isopropanol and incubated at −20 °C for one hour and then centrifuged at 12,000× *g* for 10 min. The pellet was washed with 70% ethanol and the resulting pellet was suspended with nuclease free water. The concentration of isolated DNA was quantified using a DeNOVIX, DS-11+Spectrophotometer and further stored at −20 °C for the PCR amplification process. The oligonucleotide primers namely FA2 (5´GTCTGGTGCCAGCAGCCGCG3〲) and RA2 (5´CCCGTGTTGAGTCAAATTAA G3〲) were used for the partial nuclear small subunit 18S rRNA region of the thraustochytrid gene for PCR amplification [77]. The PCR mixture (30 µL) contained 15 µL of 2 × PCR master mix, 2 µL of each primer, 1 µL of genomic DNA, and 10 µL of distilled water. PCR amplification was carried out using an Applied Bio SystemsVeritiThermal Cycler (Thermo Fisher Scientific, Waltham, MA, USA) with an initial denaturation (4 min at 94 °C), followed by 35 cycles of denaturation (1 min at 94 °C), annealing (1 min at 55 °C), extension (2 min at 72 °C), and a final extension step (10 min at 72 °C). The purity of the PCR product was checked by 1.2% agarose gel electrophoresis and visualized under a BIO RAD, Gel Doc Molecular Imager. Concentration of amplicon was checked in Nanodrop ND 8000 (DeNOVIX Inc., Wilmington, NC, USA). The PCR product was purified using a PureLink purification column (Invitrogen BioServices India Pvt. Ltd., Bangalore, India.), and further product was sequenced using the automated DNA sequencer ABI 3730xl cycle sequencer (Agri genomes, Kerala, India). Forward and reverse sequences were assembled and contig was generated after trimming the low quality bases and resultant sequences were compared with reference sequences in the bioinformatics tool BLAST of the NCBI database. The sequences were aligned with 108 18S rRNA various thraustochytrid sequences at species level retrieved from the NCBI database using the BIOEDIT software (BioEdit Sequencer Alignment Editor, Version 7.0.5.3, Scotts Valley, CA, USA) and were subsequently used for the construction of a maximum-likelihood phylogenetic tree with MEGA 6 software (MEGA Version 6, Tempe, AZ, USA).

The evolutionary history was inferred by using the maximum likelihood method based on the Tamura–Nei model [78]. The tree with the highest log likelihood (−30,824.6437) is shown. The percentage of trees in which the associated taxa clustered together is shown next to the branches. Initial tree(s) for the heuristic search were obtained by applying the neighbor-joining method to a matrix of pairwise distances estimated using the maximum composite likelihood (MCL) approach. The tree is drawn to scale, with branch lengths measured in the number of substitutions per site. The analysis includes a total of 108 18S rRNA nucleotide sequences belonging to different genera including sequences obtained in the present study. There were a total of 2149 positions in the final dataset. Evolutionary analyses were conducted in MEGA6 [79] and bootstrap support was evaluated with 1000 replicates. All 18S rRNA sequences were submitted to the NCBI GenBank and phylogenetic analysis was carried out to determine the relationship between the Andaman isolates and other members of thraustochytriaceae. 

### 3.6. Effect of Temperature, Salinity, and pH Tolerance

Thraustochytrid strains were subjected to various conditions of temperature, salinity, and pH to identify their stress tolerance. The pure culture was screened for salinity tolerance by growing in GYP broth media at different salinity ranges from 0 to 100 PSU at 5 PSU interval differences by altering synthetic sea salt by maintaining a temperature at 30 °C and pH of 7.2. Tolerance of the pH test was done by growing the cultures in GYP broth media with different pH ranges from 4 to 11, adjusted with 0.1 M HCl and 0.1 M NaOH at the adopted temperature of 30 °C and salinity of 30 PSU. Additionally, the temperature tolerance of the isolates were studied by growing them in GYPA in different temperature ranges of 5, 10, 15, 20, 25, 30, 35, 40, 45, and 50 °C and maintaining a pH of 7.2 and salinity of 30 PSU.

### 3.7. Observation of Thraustochytrids Growth Behavior 

A loop of pure thraustochytrid cell culture was inoculated in freshly prepared GYP broth (glucose 3 g·L^−1^, yeast extract 1.25 g·L^−1^, and peptone 1.25 g·L^−1^, with the addition of antibacterial streptomycin 100 µg·L^−1^ and antifungal fluconazole 100 µg·L^−1^ agents to prevent contaminations, 7.2 pH, and 28 PSU salinity) in a 1000 mL Erlenmeyer flask and incubated at 28 °C, with constant rotary shaking at 120× g (Orbitek Shaker, Scigenics Biotech, Chennai, Tamil Nadu, India). This experimental study was conducted for up to nine days for growth pattern studies. The growth patterns of selected thraustochytrids were plotted by measuring the optical density (OD) at 600 nm (PerkinElmer, UV–VIS Spectrometer, Lambda 25, Waltham, MA, USA) for up to 196 h. The growth behavior of thraustochytrids at different time intervals was studied in triplicate.

### 3.8. Biomass Production of Thraustochytrids

Thraustochytrids were cultured separately in production broth medium containing modified glucose (10 g·L^−1^), yeast (3 g·L^−1^), and peptone (5 g·L^−1^) (MGYP) prepared in seawater for biomass production. The cultures were grown in a 5000 mL flask filled with 1000 mL culture medium under a 150× *g* shaking condition (Orbitek Shaker, Scigenics Biotech, Chennai, Tamil Nadu, India) at 28 °C for five to seven days. Each culture flask was plugged with sterile cotton to avoid contamination. Biomass of thraustochytrids was harvested through the centrifugation method at 8000× *g* for 5 min using a conical bottom centrifuge tube (50 mL, Himedia). The cells were washed twice with sterile distilled water to remove the medium component from the biomass. Cell samples were then freeze-dried (−40 °C) for 48 h and weighed. Biomass production was estimated as gram of dry weight biomass per one liter of growth medium. Biomass was stored in sealed containers at −80 °C for further studies.

### 3.9. Lipid Extraction and Fatty Acid Methyl Ester Analysis

The total lipids from the lyophilized cells were extracted using chloroform: methanol (2:1 ratio) by adopting the methods of Floch et al. (1957) [80]. The total lipids were transferred to an air-tight vial and allowed to dry free from the solvent using nitrogen gas. The dry lipid was added with 1 mL of 3% methanolic:HCl and incubated at 60 °C for 18 h for the trans-esterification reaction by adopting the method of Kashiwagi et al. (1997) [81]. The upper organic phase was carefully transferred to a chromatographic vial and evaporated to dryness using nitrogen gas and then 1 mL of ethyl acetate was added to the content of the vial and analyzed for FAME. FAME was analyzed through GC-MS (Agilent 7890A-240 MS with Ion Trap, Santa Clara, CA, USA). The GC-MS was fitted with a silica capillary column Agilent J&W, HP-5ms of 30 m × 0.250 mm × 0.25 µm (Agilent Technologies, Santa Clara, CA, USA) connected to MSD. The carrier gas used was helium (1 mL/min) and the mobile phase was nitrogen (35 mL/min). The oven temperature was programmed with an initial temperature of 140 °C at 1 min, increased at a rate of 2 °C/min to achieve the final temperature of 220 °C, and was held for 1 min, Then the injector temperature was set to 260 °C, sample volume of 1 μL, and the split ratio at 100:1. An external standard mix (FAME Mix C4–C24, Sigma Aldrich, Burlington, MA, USA) was used to standardize the sample's composition of fatty acid methyl esters. The FAME was also identified using the built-in NIST library in the software.

### 3.10. Blood Agar Hemolysis Activity

Toxicity of thraustochytrid strains was tested by the hemolysis assay [82]. The blood agar (Himedia bioscience, India) was prepared in sterile seawater. After autoclaving, the medium was cooled to 40–50 °C, where 5% sterile defibrinated sheep blood was added, mixed well gently, the final pH was adjusted to 7.3, and then poured into sterile Petri plates. The fresh culture of thraustochytrids was inoculated into the blood agar and plates were incubated for 24 to 48 h at 30 °C. Blood agar lysis was detected by measuring the zone of hemolysis around thraustochytrids in culture plates. 

### 3.11. Screening of Antibacterial Activity

The antibacterial activity of the crude extract of thraustochytrids was tested against human and fish bacterial pathogenic strains such as *Escherichia coli (E. coli), Staphylococcus aureus (S. aureus), Vibrio cholera (V. cholera), Klebsilla pneumonia (K. pneumonia),* and *Bacillus subtilis (B. subtilis),* obtained from microbial culture maintenance laboratory, Department of Medical Microbiology, Rajah Muthaiah Medical College, Annamalai University, Tamil Nadu, India whereas fish pathogenic strains such as *Vibrio harveyi* *(V. harveyi)* and *Vibrio parahaemolyticus* *(V. parahaemolyticus)*, *Vibrio alginolyticus* (*V. alginolyticus*) and *Vibrio campilli* (*V. campilli*) were obtained from the Rajiv Gandhi Center for Aquaculture, Nagapattinam District, Tamil Nadu, and India. Briefly, the freeze-dried cells of 1000 mg were extracted with petroleum ether in Soxhlet apparatus. The extract was filtered using Whatman No. 1 filter papers and then evaporated in a rotary vacuum evaporator. The antibacterial activity of thraustochytrid crude extracts was screened by the disc diffusion method [22,25,83]. The 100 µL of 24 h old cell culture of human and fish pathogens was poured on to the plates prepared in Muller–Hinton agar thinly over the plate by the spread plate method using a glass L rod aseptically. For the disc diffusion assay, 50 µL of the extract was loaded onto a 6 mm paper disc (Himedia, Mumbai, India) and allowed to dry completely. The disc was then placed on the surface of the freshly inoculated agar plates. The plate was incubated at 30 °C for 24 to 48 h and the antibacterial activity was evaluated by measuring the diameter of the inhibition zone around the disc. Ampicillin was used as a positive antibiotic control. All experiments were performed in triplicate.

### 3.12. Antioxidant Activity

The DPPH free radical scavenging activity of the thraustochytrid extract was estimated using the method of Duan et al. [84] with modifications. The lyophilized thraustochytrid biomass (200 mg) was extracted in 4 mL of methanol and prepared in four different concentrations (100, 200, 300, 400, and 500 μg·mL^−1^). About 4 mL of DPPH (5 mg DPPH in 2 mL of ethanol) solution was mixed with different concentrations of thraustochytrid extracts. The samples were shaken well and all test tubes were kept in the dark for 45 min at room temperature. The absorbance of the reaction mixture was measured at 517 nm using a spectrophotometer (PerkinElmer, UV–VIS Spectrometer, Lambda 25, Waltham, MA, USA). The control was prepared in the same way without adding any crude extract. L-Ascorbic acid was used as the positive control. Each experiment was conducted in triplicate. The percentage of free radical scavenging activity was calculated by using the formula and is expressed in percentage.

% activity = [(control absorbance (Ac) − extract absorbance (As)/(control absorbance (Ac)] ×100(1)

where Ac = control, and As = sample.

## 4. Conclusions

This is the first detailed study of thraustochytrids for their diversity, fatty acid profile, and biological activities in south Andaman mangrove habitats. Two major thraustochytrid genera (*Thraustochytrium, Aurantiochytrium*) were recorded with morphological and molecular information. The thraustochytrid strains showed good tolerance with pH, temperature, and salinity. The highest production of biomass (ANVKK-06), lipid (ANVKK-03), DHA (ANVKK-06), and EPA (ANVKK-04) was recorded. ANVKK-03 and ANVKK-10 displayed antibacterial activity in terms of the maximum zone of inhibition against human and fish pathogens of *S. aureus* and *V. parahaemolyticus.* ANVKK-06 showed antioxidant activity in terms of the highest free radical scavenging activity. Thraustochytrid strains were proven to be non-toxic as evident by negative blood agar hemolysis. *Aurantiochytrium* sp., (ANVKK-06), was found to have the potential for future bio-prospecting potential.

## Figures and Tables

**Table 1:** Morphological features of the newly isolated thraustochytrids.

Isolate No.	Mangrove Habitats	Colony Color	Colony Morphology	Ectoplasmic Network	Cell Wall	Amoeboid Cell	Binary Cell Division	Organism Name
ANVKK-01	MB	Pale white	Large size colonies, binary cell partition, thin wall globes, or spherical shape of thallus. 2, 4, 8 cluster of vegetative cells, biflagellate zoospores	Well developed	Thin cell wall	Amoeboid cell present	Present	*Aurantiochytrium* sp.
ANVKK-02	CC	Pale white	Medium size colonies, oblong or sub-oblong shape cells, amoeboid cells present, internal proliferation with motile zoospore formation, presence of biflagellate zoospores	Absent	Thick cell wall	Amoeboid cell absent	Absent	*Thraustochytrium* sp.
ANVKK-03	MJ, MB	Orange	Moderate size colonies, globes and sub globes thallus. Amoeboid cells present, binary cell division present	Well developed	Thin cell wall	Amoeboid cell present	Present	*Aurantiochytrium limacinum.*
ANVKK-04	GP	White	Medium size colonies, bi-partition cleavage, diads, tetrads, and different numbers vegetative cells present, present, globes, spherical, or pseudopodia thallus, reniform or ovoid shape zoospores	Well developed	Thin cell wall	Amoeboid cell present	Present	*Aurantiochytrium* sp.
ANVKK-05	BN	White	Large size colonies, spherical, or sub-spherical shape thallus, internal proliferation with motile zoospore formation, biflagellate zoospores present	Well developed	Thin cell wall	Amoeboid cell present	Absent	*Thraustochytriidae* sp.
ANVKK-06	SG	Creamy orange	Small to large size colonies, globes and sub-globes thallus, binary cell division present, 2, 4, 7, and different numbers of vegetative cells present, Limaciform amoeboid cells present	Well developed	Thin cell wall	Amoeboid cell present	Present	*Aurantiochytrium* sp.
ANVKK-07	CT	Creamy white	Small size colonies, globes and sub-globes thallus, multiproliferous bodies, Zoosporangia contain large vesicles	Absent	Thick cell wall	Amoeboid cell present	Absent	*Aurantiochytrium* sp.
ANVKK-08	WD	Creamy white	Moderate to very large size colonies, binary cell division present, 2, 4, and different numbers of vegetative cells present, Pseudopodia or spherical shape of cell, zoospores actively present	Un-developed	Thin cell wall	Amoeboid cell present	Present	*Aurantiochytrium* sp.
ANVKK-09	CC	Pale white	Medium size colonies, globes or sub-globes, bi-partition cleavage, tetrads, ovoid shape zoospores	Well developed	Thin cell wall	Amoeboid cell present	Present	*Aurantiochytrium* sp.
ANVKK-10	MJ	Creamy white	Moderate size colonies, large vesicle, multiproliferous bodies, zoosporangia contain large vesicle, many active zoospores	Un-developed	Thick cell wall	Amoeboid cell present	Absent	*Aurantiochytrium* sp.
ANVKK-11	CC	Pale white	Small to medium, globes, or sub-globes shape of cell, binary cell division present, diads, tetrads, and different numbers of cells present, many active amoeboid cells present	Well developed	Thin cell wall	Amoeboid cell present	Present	*Aurantiochytrium* sp.
ANVKK-12	CT	Creamy white	Medium size cell, spherical, or pseudopodia shape of cell, bi-partition cleavage, 2, 4, and different numbers of vegetative cells present, different shape of limaciform amoeboid cells present	Well developed	Thin cell wall	Amoeboid cell present	Present	*Aurantiochytrium* sp.

MB—Minibay, CC—Carbyns cove, BN—Burmanallah, SG—Sippighat, CT—Chidiyatapu, WD—Wandoor, GP—Guptapara, MJ—Manjery.

**Table 2:** Physical parameter tolerances ranges and blood agar hemolysis.

Isolates.	Growth Range of pH Tolerances	Optimum pH	Growth Range of Temperature Tolerances	Optimum Temperature	Growth range of Salinity Tolerances	Optimum Salinity	Blood Agar Hemolysis
ANVKK-01	4–10	6.5–7.5	10–35	25–30	0–100	25–30	Negative
ANVKK-02	4–10	6.5–7.5	10–35	25–30	0–100	25–30	Negative
ANVKK-03	5–9	6.5–7.5	15–35	25–30	5–90	25–30	Negative
ANVKK-04	4–10	6.5–7.5	10–35	25–30	0–100	25–30	Negative
ANVKK-05	4–10	6.5–7.5	10–35	25–30	0–100	25–30	Negative
ANVKK-06	4–10	6.5–7.5	10–35	25–30	0–100	25–30	Negative
ANVKK-07	4–10	6.5–7.5	10–35	25–30	0–100	25–30	Negative
ANVKK-08	4–10	6.5–7.5	10–35	25–30	5–90	25–30	Negative
ANVKK-09	4–10	6.5–7.5	10–35	25–30	0–100	25–30	Negative
ANVKK-10	5–9	6.5–7.5	10–35	25–30	5–90	25–30	Negative
ANVKK-11	4–10	6.5–7.5	10–35	25–30	0–100	25–30	Negative
ANVKK-12	4–10	6.5–7.5	10–35	25–30	0–100	25–30	Negative

The optimal culture conditions for screening the physical parameter tolerances were found to be 3 g·L^−1^ of glucose, 1.25 g·L^−1^ of yeast extract, and 1.25 g·L^−1^ of peptone, 7.2 pH, 28 psu salinity, 28 °C temperature.

**Table 3:** Fatty acid composition (expressed as % of the total fatty acids) of thraustochytrids isolates.

Fatty Acids	ANVKK-01	ANVKK-02	ANVKK-03	ANVKK-04	ANVKK-05	ANVKK-06	ANVKK-07	ANVKK-08	ANVKK-09	ANVKK-10	ANVKK-11	ANVKK-12
C4:0	0.24	0	0	0	0	0	0	0.07	0.64	0.01	0.56	0
C7:0	0.04	0	0	0	0	0	0	0.12	0.5	0	0	0.14
C11:0	0.50	0	0	0.23	3.31	0.15	0	1.46	1.21	0.3	0.93	0.12
C12:0	0.41	0	0	0.61	2.28	0.19	0.21	2.17	0.43	0.02	0.61	0.64
C13:0	6.81	0	0	0.09	7.03	2.04	2.23	5.33	6.54	0.43	6.05	8.02
C14:0	12.62	27.59	0	2.59	1.84	0.68	1.37	8.45	5.39	8.68	3.87	2.23
C14:1	2.4	0	0	0.11	11.28	5.37	10.44	0.35	2.64	0.03	9.12	0.26
C15:0	0	0	0	18.02	29.1	2.23	16.63	29.56	28.77	0.74	0	0
C16:0	46.11	39.71	41.05	8.32	3.18	19.63	1.39	0.13	6.55	28.17	27.76	32.76
C16:1	5.33	14.11	23.68	0.38	4.15	5.04	7.55	6.67	4.63	1.55	2.53	0.82
C16:2	0.26	0	0	1.15	8.75	0.16	0.03	0.04	0	0.17	0	0.43
C16:3	0.52	0	0	0.08	0	0.06	0	0	0.42	0.07	1.38	0
C16:4	0	0	0	0	0	0.42	0	0	0	0	0	0
C17:1	2.41	8.56	1.11	0.07	0	1.78	4.53	0.79	4.78	0.56	3.56	3.89
C17:3	0.44	0	0.34	0.98	0	0.21	0.08	0.17	0.08	0.11	0.76	0.38
C18:0	0.86	0	0	0	0	0	2.13	3.32	0	2.56	0	1.61
C18:1	1.55	0.98	1.51	2.06	3.61	0.09	0.22	0.39	0.1	0.14	3.41	0.21
C18:2	0	0	0	0.16	0.78	0.07	0	0	0	1.08	0	0.29
C18:3	0	0	0	0	0	2.05	2.41	0	1.03	0	0.98	1.3
C20:0	2.34	5.69	0	0.98	1.21	0.26	0.34	1.08	0.67	0.65	2.59	3.64
C20:3	0.28	0.23	0	2.47	4.67	0.08	0.65	0	0	0.34	0	0.33
C20:4	1.04	0	0.44	0.34	0	0.4	0.75	3.92	0.51	0.87	2.61	3.94
C20:5	0.34	0	0.37	11.03	0	3.67	1.91	8.16	3.2	4.41	0.98	6.92
C21:5	0.56	0	0.24	1.61	0	0.02	0.19	0	0	0.08	0	0
C22:5	2.91	0.76	1.07	4.65	2.73	8.21	8.65	5.16	8.01	6.89	5.67	0
C22:6	12.98	2.37	30.19	44.07	16.08	47.19	38.29	22.66	23.9	42.14	26.63	32.07

The optimal culture conditions for fatty acid production were observed at 7.2 pH, 28 psu salinity, 28 °C temperature, fourth day of incubation, 10 g·L^−1^ of glucose, 3 g·L^−1^ of yeast extract, and 5 g·L^−1^ of peptone and biomass was freeze-dried at −40 °C for 48 h. The highest percent of omega-3 fatty acids, DHA (47.19%) were recorded in ANVKK-06 at 96 h.

**Table 4:** Antibacterial activity of thraustochytrid isolates crude extracts against human and fish bacterial pathogens.

Clinical Pathogens	Zone of Inhibition (mm) (Mean ± S.D)												
Human Bacterial Pathogens	01	02	03	04	05	06	07	08	09	10	11	12	A (C)
*E. coli*	6.32 ± 0.5	11.03 ± 0.4	7.81 ± 1.0	-	-	5.96 ± 1.52	4.34 ± 0.5	12.55 ± 0.6	-	11.06 ± 0.7	9.10 ± 1.0	16.63 ± 1.5	22.11 ± 0.5
*S. aureus*	8.06 ± 0.3	-	18.69 ± 1.2	12.58 ± 0.4	9.11 ± 0.2	17.58 ± 0.6	11.32 ± 1.5	-	15 ± 1.5	6.19 ± 1.15	16.24 ± 1.5	10.03 ± 0.5	20.27 ± 0.8
*K. pneumonia*	-	12.10 ± 0.6	-	11.73 ± 1.5	14.33 ± 1.1	-	-	11.45 ± 1.0	16.34 ± 0.7	5.57 ± 1.5	-	4.56 ± 1.5	21.09 ± 0.4
*V. cholera*	4.32 ± 0.2	-	13.06 ± 0.5	-	7.89 ± 0.3	5.03 ± 1.5	14 ± 1.2	-	7.08 ± 0.2	10.56 ± 0.5	7.35 ± 0.8	-	19.21 ± 1.5
*B. subtilis*	-	8.00 ± 0.7	13.66 ± 1.5	10.97 ± 0.2	-	17.66 ± 0.5	15.23 ± 0.2	14.11 ± 2.0	-	11 ± 1.0	14.51 ± 1.5	11.40 ± 1.0	21.12 ± 0.5
**Fish Pathogens**													
*V. alginolyticus*	9.02 ± 0.8	4.15 ± 0.4	4.67 ± 1.52	9.24 ± 0.3	-	7.23 ± 0.51	11.87 ± 2.0	-	14.33 ± 0.5	-	6.79 ± 1.15	-	16.34 ± 2.0
*V. parahaemolyticus*	-	7.33 ± 1.1	-	17.00 ± 1.5	7.66 ± 0.5	7.89 ± 0.55	-	9.33 ± 0.6	7.63 ± 0.3	18.31 ± 1.0	-	10.34 ± 0.5	21.23 ± 0.5
*V. campilli*	14.58 ± 0.4	-	9.03 ± 0.57	10.87 ± 1.3	5.56 ± 1.12	-	15.33 ± 1.5	10.66 ± 0.5	-	7.78 ± 1.52	5.78 ± 2.0	7.33 ± 1.5	18 ± 1.52
*V. harveyi*	-	14.67 ± 0.5	8.97 ± 0.6	-	-	5.09 ± 1.5	9.87 ± 1.1	-	5.78 ± 1.5	16.66 ± 1.1	11 ± 0.5	4.66 ± 1.12	19 ± 0.58

1—ANVKK-01, 2—ANVKK-02, 3—ANVKK-03, 4—ANVKK-04, 5—ANVKK-05, 6—ANVKK-06, 7—ANVKK-07, 8—ANVKK-08, 9—ANVKK-09, 10—ANVKK-10, 11—ANVKK-11, 12—ANVKK-12, and A—Positive control (Ampicillin).

## Data Availability

Not applicable.

## Supplementary Materials

The following are available online at https://www.mdpi.com/article/10.3390/md19100571/s1, Figure S1. (a) Morphological, (b) light, and (c) scanning electron microscopy of ANVKK-01 isolates, Figure S2. (a) Morphological, (b) light, and (c) scanning electron microscopy of ANVKK-02 isolates, Figure S3. (a) Morphological, (b) light, and (c) scanning electron microscopy of ANVKK-03 isolates, Figure S4. (a) Morphological, (b) light, and (c) scanning electron microscopy of ANVKK-04 isolates, Figure S5. (a) Morphological, (b) light, and (c) scanning electron microscopy of ANVKK-05 isolates, Figure S6. (a) Morphological, (b) light, and (c) scanning electron microscopy of ANVKK-06 isolates, Figure S7. (a) Morphological, (b) light, and (c) scanning electron microscopy of ANVKK-07 isolates, Figure S8. (a) Morphological, (b) light, and (c) scanning electron microscopy of ANVKK-08 isolates, Figure S9. (a) Morphological, (b) light, and (c) scanning electron microscopy of ANVKK-09 isolates, Figure S10. (a) Morphological, (b) light, and (c) scanning electron microscopy of ANVKK-10 isolates, Figure S11. (a) Morphological, (b) light, and (c) scanning electron microscopy of ANVKK-11 isolates, Figure S12. (a) Morphological, (b) light, and (c) scanning electron microscopy of ANVKK-12 isolates.
