# Supplementary material for: Thraustochytrids of Mangrove Habitats from Andaman Islands: Species Diversity, PUFA Profiles and Biotechnological Potential
Source: Mar Drugs. 2021 Oct 14;19(10):571. doi: 10.3390/md19100571 (PMC8539084; doi:10.3390/md19100571)
Supplement: Supplementary file 1 [file marinedrugs-19-00571-s001.zip › marinedrugs-1353009 -si-revised.pdf]

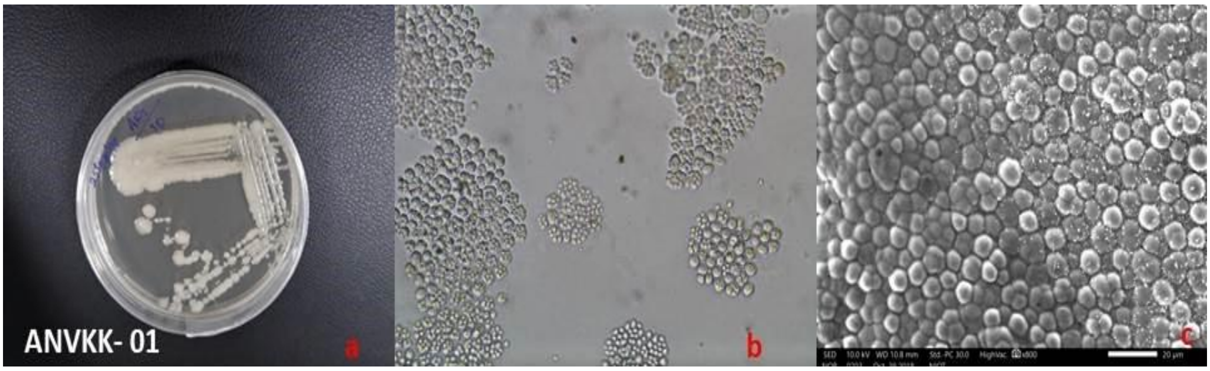

Supplementary Figure S1. (a) Morphological, (b) light, and (c) scanning electron microscopy of ANVKK-01 isolates

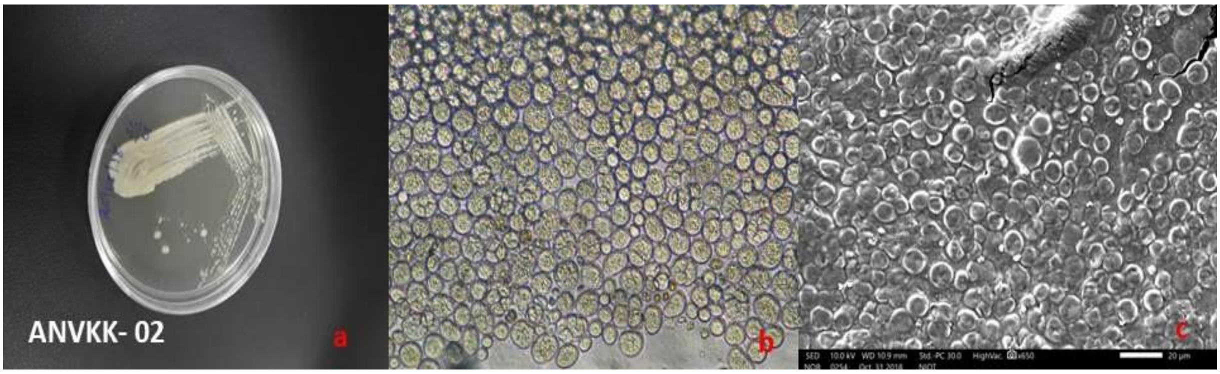

Supplementary Figure S2. (a) Morphological, (b) light, and (c) scanning electron microscopy of ANVKK-02 isolates

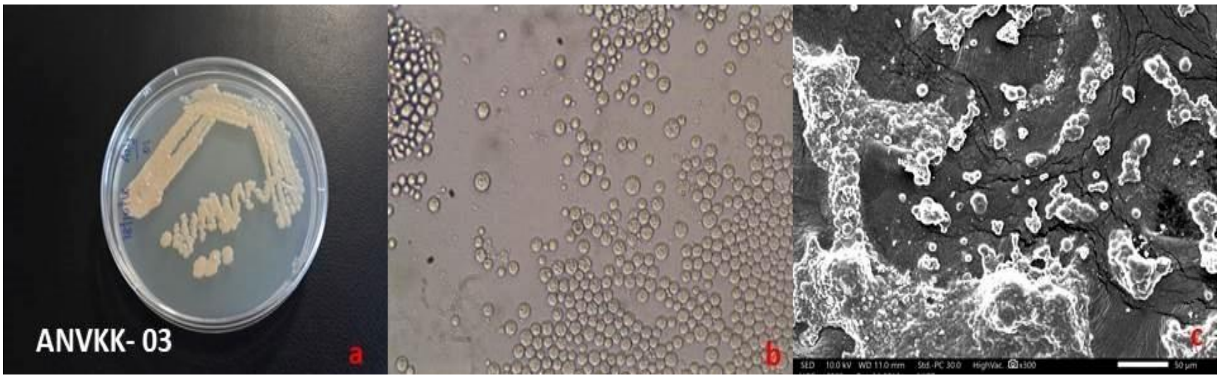

Supplementary Figure S3. (a) Morphological, (b) light, and (c) scanning electron microscopy of ANVKK-03 isolates

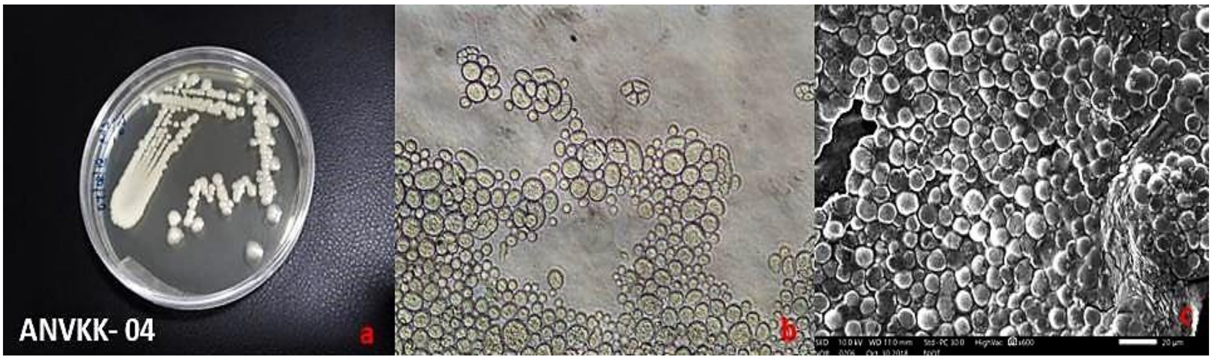

Supplementary Figure S4. (a) Morphological, (b) light, and (c) scanning electron microscopy of ANVKK-04 isolates

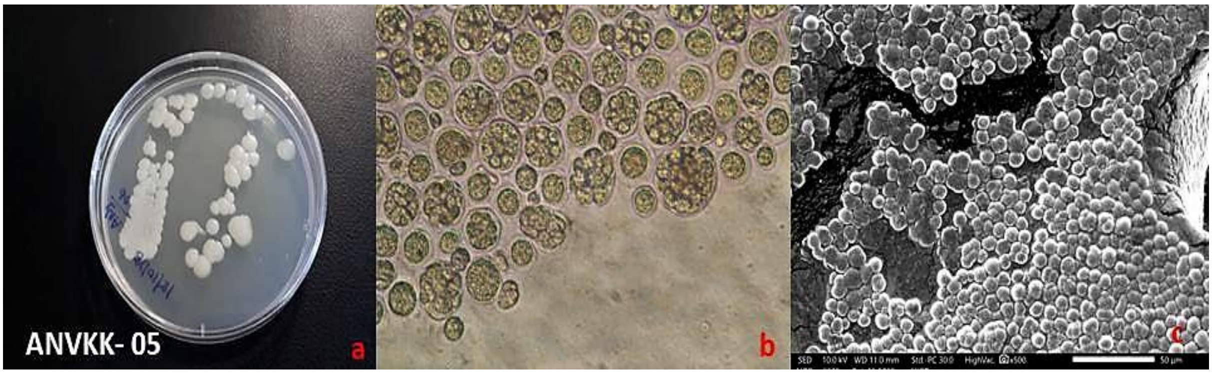

Supplementary Figure S5. (a) Morphological, (b) light, and (c) scanning electron microscopy of ANVKK-05 isolates

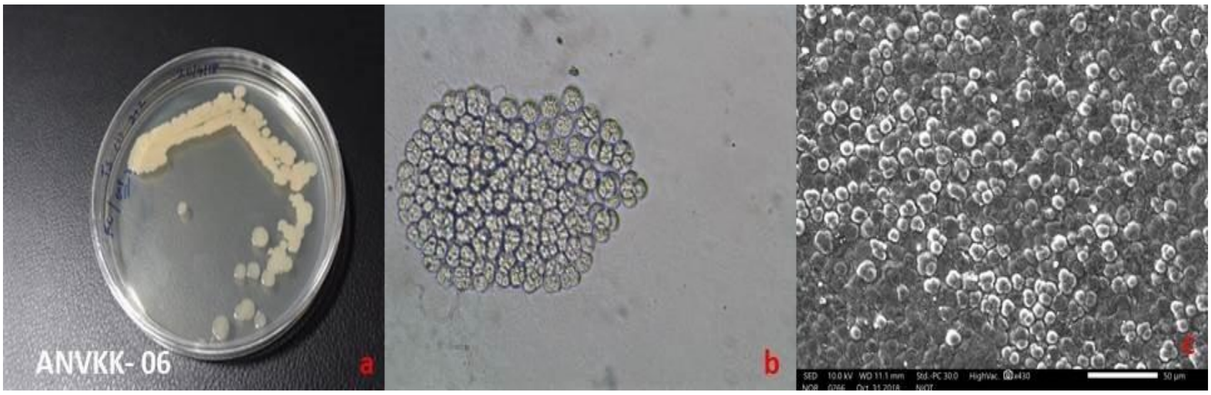

Supplementary Figure S6. (a) Morphological, (b) light, and (c) scanning electron microscopy of ANVKK-06 isolates

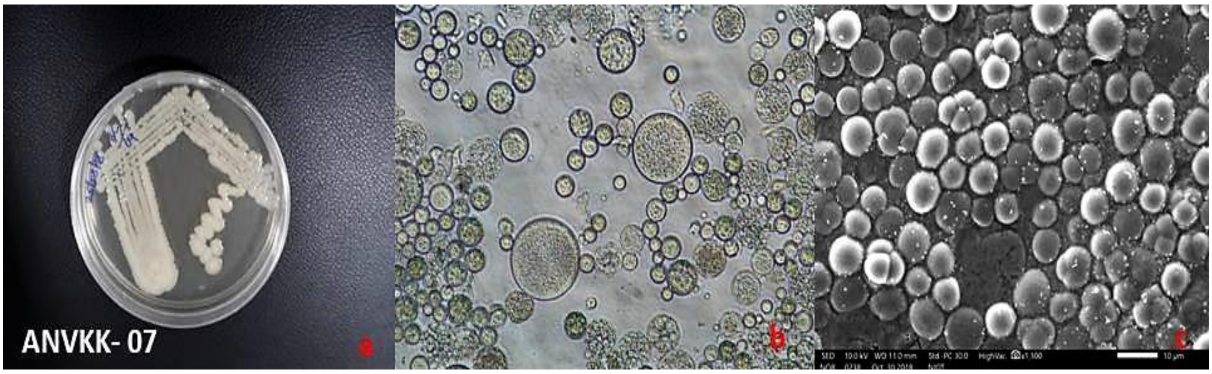

Supplementary Figure S7. (a) Morphological, (b) light, and (c) scanning electron microscopy of ANVKK-07 isolates

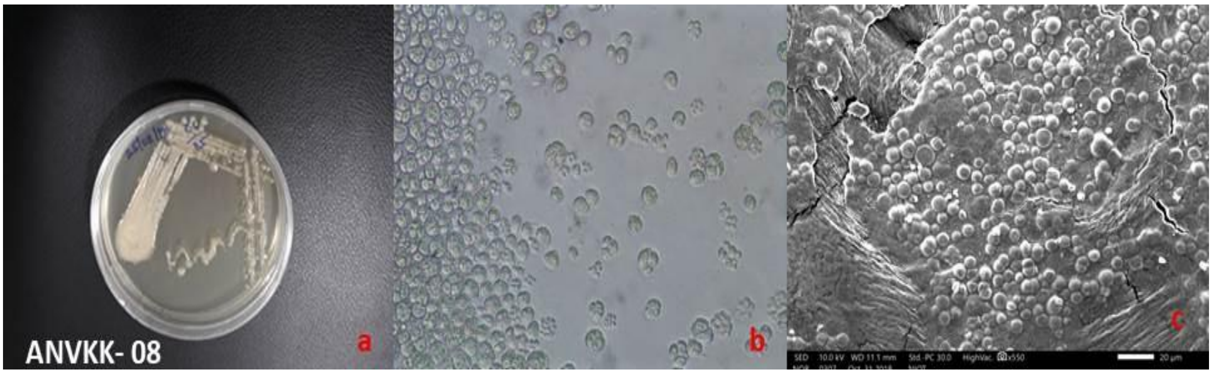

Supplementary Figure S8. (a) Morphological, (b) light, and (c) scanning electron microscopy of ANVKK-08 isolates

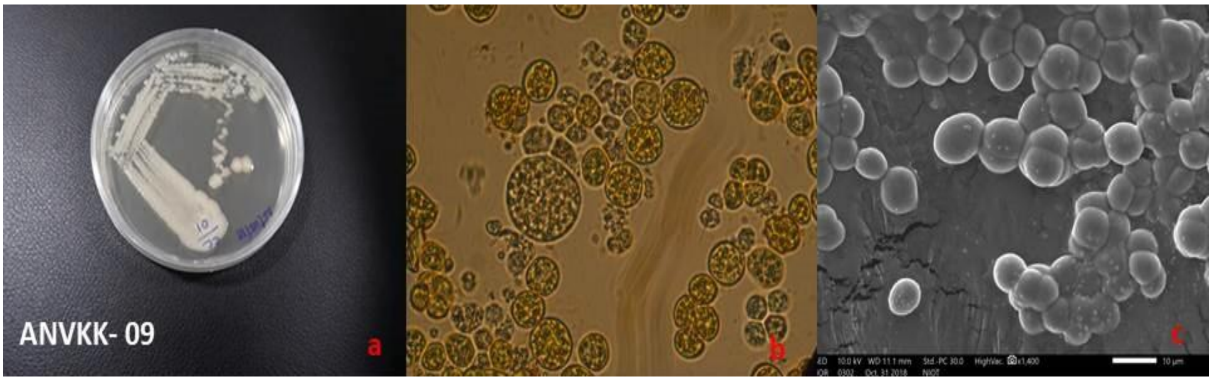

Supplementary Figure S9. (a) Morphological, (b) light, and (c) scanning electron microscopy of ANVKK-09 isolates

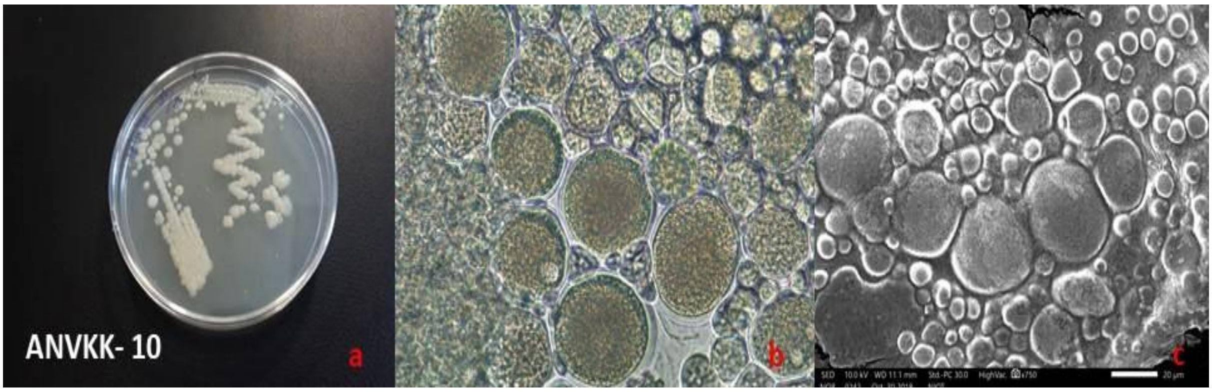

Supplementary Figure S10. (a) Morphological, (b) light, and (c) scanning electron microscopy of ANVKK-10 isolates

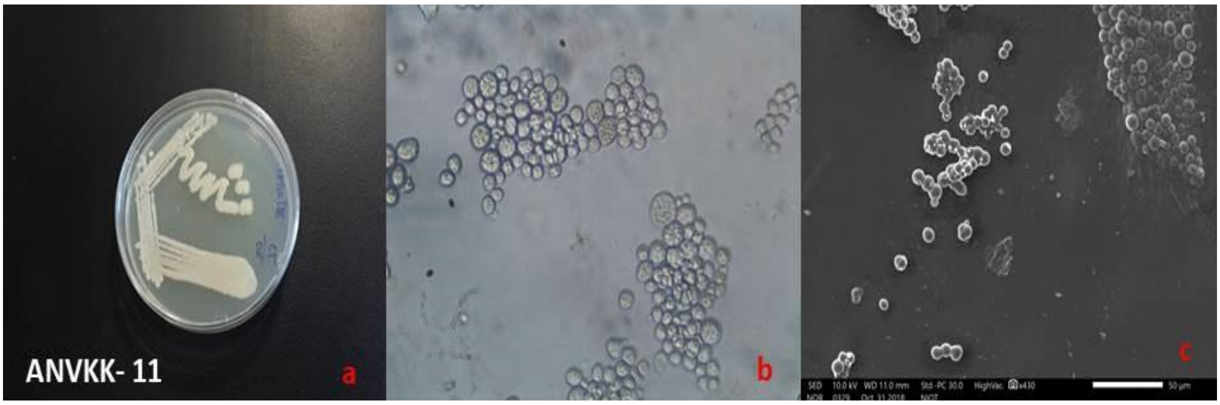

Supplementary Figure S11. (a) Morphological, (b) light, and (c) scanning electron microscopy of ANVKK-11 isolates

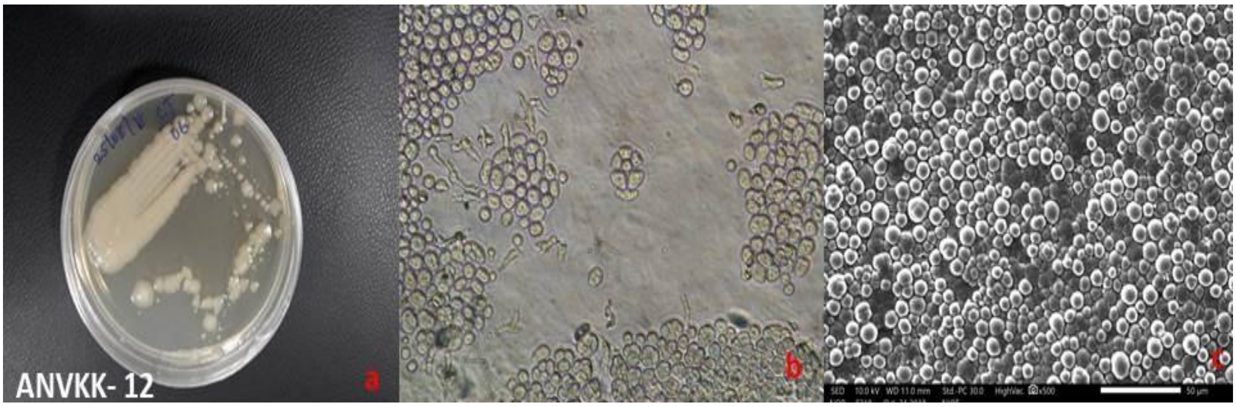

Supplementary Figure S12. (a) Morphological, (b) light, and (c) scanning electron microscopy of ANVKK-12 isolates
